# Successful Surgical Treatment of a Rare Case of Acute Isolated Right Ventricle Wall Rupture Caused by Distal Circumflex Coronary Artery Occlusion

**DOI:** 10.21470/1678-9741-2020-0530

**Published:** 2022

**Authors:** Emrah Erdogan, Yakup Kilic, Hasim Tuner, Sahin Sahinalp, Anantharaman Ramasamy

**Affiliations:** 1 Department of Cardiology, Barts Heart Centre, Barts Health NHS Trust, London, UK.; 2 Department of Cardiovascular Surgery, Van Yuzuncuyil University Hospital, Van, Turkey.

**Keywords:** Myocardial Infarction, Echocardiography, Cardiogenic Shock, Heart Rupture, Vascular Diseases.

## Abstract

We describe a rare case of isolated right ventricular inferior free-wall rupture and cardiogenic shock caused by occlusion of the distal left circumflex coronary artery. Our case highlights the central role of transthoracic echocardiography in identifying unexpected conditions that can guide management - in our case involving early surgical intervention, thus leading to favourable patient outcomes.

**Table:** 

Abbreviations, acronyms & symbols	
ECG	= Electrocardiogram
RV FWR	= Right ventricular free-wall rupture
TTE	= Transthoracic echocardiogram

## INTRODUCTION

Ventricular free-wall rupture is a well-known complication after myocardial infarction, associated with an overall incidence of 6.2%^[1]^. The most common presentation is with cardiac arrest and tamponade. However, due to advances in pharmacotherapy and coronary intervention, studies have shown a reduction in the overall incidence of free-wall rupture^[2]^. Our case is unique from a coronary point of view as it is the only case of isolated right ventricular free-wall rupture caused by distal circumflex coronary artery occlusion.

### History of Presentation

A 70-year-old male presented to the emergency department with progessively worsening chest pain, palpitations and shortness of breath for four hours. In the past three months, he complained of exertional chest pain that settled at rest and was compliant with his anti-anginal medications. His observations were significant for blood pressure 70/30 mmHg and a sinus tachycardia of 125 beats per minute. On examination he was confused and had signs of poor peripheral perfusion with cool lower extremities, elevated jugular venous pressure, muffled heart sounds and pulsus paradoxus. The patient had known hypertension of which he was on losartan 50 mg once a day. He had a history of heavy smoking for the past 40 years and did not have previous coronary intervention or cardiac surgery.

**Fig. 1:**
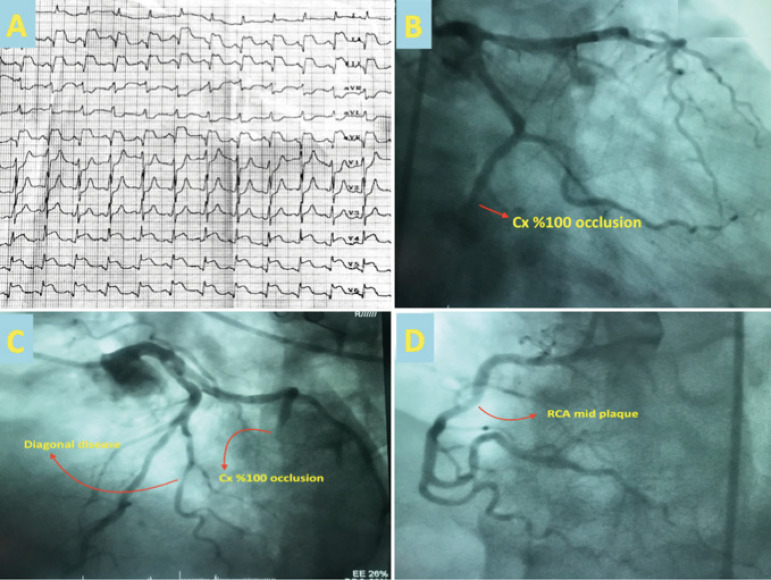
Serum RhoA levels of the groups. CAD=coronary artery disease.

**Fig. 2:**
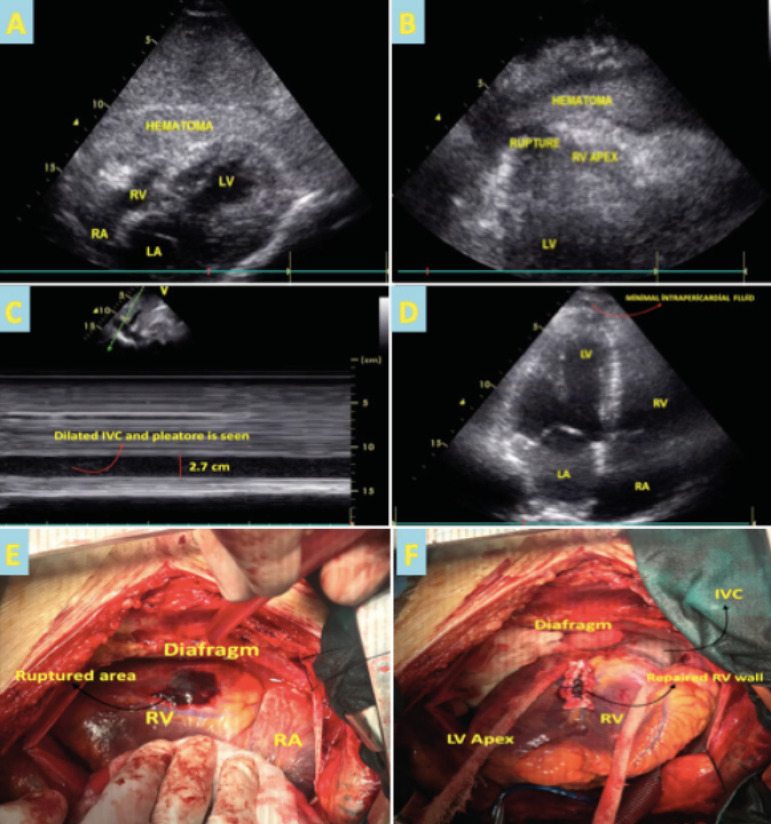
Serum RhoA levels of the groups. CAD=coronary artery disease.

An initial electrocardiogram (ECG) revealed inferolateral ST segment elevation (Figure 1A). Significant blood test findings were a raised troponin T of 2,000 ng/L (TnT 14-30 ng/L), negative inflammatory markers (C-reactive protein 0.5 mg/dl, white blood cells 6.0 × 10^3^/ul) and normal kidney function. Given the patient’s unstable hemodynamic state, an immediate bedside transthoracic echocardiogram (TTE) was performed that showed inferior posterior wall hypokinesia, an extensive echo-dense pericardial effusion with right ventricular collapse and inferior vena cava pleathore was observed (Figures 2A to D and Video 1). This prompted an emergency coronary angiogram that demonstrated occlusion of the distal circumlex artery and moderate disease of the diagonal artery (Figures 1B and 1C). Valvular pathology was not seen on TTE. Cardio-thoracic surgery was then consulted as findings of the TTE raised suspicion of right ventricular free-wall rupture (RV FWR) resulting in cardiac tamponade, thereby explaining the elevated troponin and patient symptoms.

**Movies 1 and 2:**
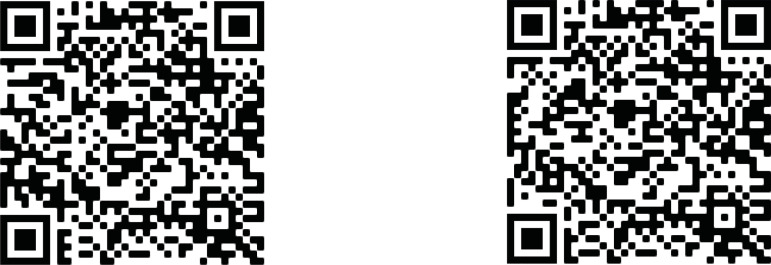

The patient was initially hemodynamically stabilised with noadrenaline and dopamine. We sought an urgent cardiothoracic surgery consultation that concluded with emergency surgery given the patient’s persistant hypotension, TTE findings and only distal circumflex occlusion on angiogram not amenable to percutaneous intervention. During surgery, after median sternotomy, a significant amount of pericardial hematoma was removed and an RV FWR was seen near the posterior interventircular sulcus (Figure 2E) and repaired by suturing with pledgets (Figure 2F). The procedure was performed on-pump in the arrested heart. Further attempt to revascularise the distal circumlex artery was not considered as most of the territory appeared to be infarcted.

The patient was extubated 24 hours after surgery and inotropic support was weaned. Post-operative TTE showed improvement of left ventricular ejection fraction to 50% with minimal pericardial effusion (Figure 2D and Video 2). The post-operative course was uneventful and the patient was discharged 8 days after surgery. At the 1-month follow-up appointment, his symptoms had completly resolved and a repeat TTE showed no further change in ejection fraction or pericardial fluid size. He remained on optimal heart failure medical therapy and will have a follow-up TTE in 12 months.

## DISCUSSION

Mechanical complications following acute myocardial infarction can lead to free-wall, papillary or interventricular septal rupture, thus carrying high mortality (40-80%) if not diagnosed and managed in a timely manner^[3]^. Echocardiography is a key tool in assessment of the heart function following myocardial injury and can help guide management. RV FWR is an uncommon finding on TTE and its assessment can be difficult. This could be due to limited views for evaluation of the right ventricle due to its crescent shape, substernal location and presence of artifacts. Consequently, definitive diagnosis sometimes can only be found on surgical exploration. In our case, TTE helped to identify the echodense fluid around the heart most consistent with hematoma (Figures 2A and 2B), thus guiding our differential for RV FWR that was confirmed on surgical exploration. Complex cases like ours emphasise the importance of a multi-disciplinary appraoch when managing high-risk patients. Through early realisation of RV FWR with the utilisation of TTE, cardio-thoracic teams were notified in a short period, thus working together for favourable patient outcomes.

According to Furukawa et al.^[4]^, RV FWR caused by left anterior descending coronary artery occlusion is associated with ventricular septal rupture. There is a case of septal and right ventricular wall rupture caused by left circumflex artery occlusion and rare cases of isolated RV free-wall rupture caused by right coronary artery occlusion^[3,5,6,7]^.

## CONCLUSION

In conclusion, this is a rare case of isolated RV FWR secondary to occlusion of the distal circumflex artery due to an inferolateral myocardial infarction that led to cardiac tamponade and was succesfully treated with emergency surgery. This case illustrates the importance of careful evaluation of TTE findings as subtle findings can help to identify correct diagnosis and guide management. This case also highlight the need to keep in mind the possibility of solitary right ventricular free wall rupture as a mechanical complication of inferolateral acute myocardial infarction.

**Table:** 

Authors' roles & responsibilities	
YK	Substantial contributions to the conception or design of the work; or the acquisition, analysis or interpretation of data for the work; drafting the work or revising it critically for important intellectual content; final approval of the version to be published
EE	Substantial contributions to the conception or design of the work; or the acquisition, analysis or interpretation of data for the work; drafting the work or revising it critically for important intellectual content; final approval of the version to be published
HT	Substantial contributions to the conception or design of the work; or the acquisition, analysis or interpretation of data for the work; final approval of the version to be published
SS	Substantial contributions to the conception or design of the work; or the acquisition, analysis or interpretation of data for the work; final approval of the version to be published
AR	Final approval of the version to be published
